# Supplementary material for: Genome-Wide Identification and Expression Analysis of Kiwifruit Leucine-Rich Repeat Receptor-Like Proteins Reveal Their Roles in Biotic and Abiotic Stress Responses
Source: Int J Mol Sci. 2024 Apr 19;25(8):4497. doi: 10.3390/ijms25084497 (PMC11050117; doi:10.3390/ijms25084497)
Supplement: Supplementary file 1 [file ijms-25-04497-s001.zip › Supplementary_Figure.pdf]

SUPPLEMENTARY FIGURES

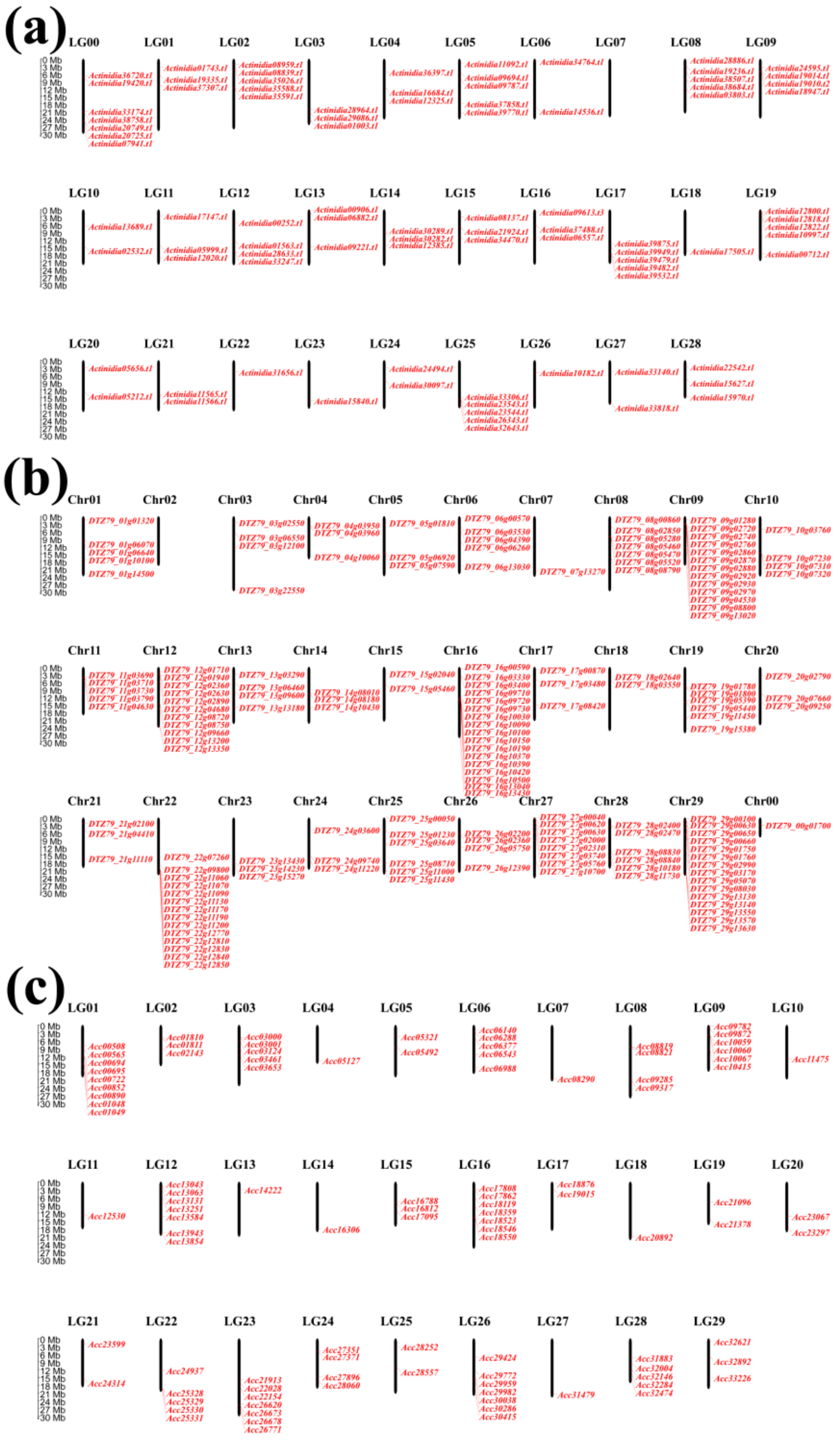

Supplementary Figure S1. Chromosomal location of *LRR-RLP* genes in kiwifruit.

(**a**) Chromosomal location of *LRR-RLP* genes in *Actinidia chinensis* ‘Hongyang’. (**b**) Chromosomal location of *LRR-RLP* genes in *Actinidia eriantha* ‘Huate’. (**c**) Chromosomal location of *LRR-RLP* genes in *Actinidia chinensis* ‘Red5’.
